# Supplementary material for: Repetitive Transcranial Magnetic Stimulation Reduces Depressive-like Behaviors, Modifies Dendritic Plasticity, and Generates Global Epigenetic Changes in the Frontal Cortex and Hippocampus in a Rodent Model of Chronic Stress
Source: Cells. 2023 Aug 14;12(16):2062. doi: 10.3390/cells12162062 (PMC10453847; doi:10.3390/cells12162062)
Supplement: Supplementary file 1 [file cells-12-02062-s001.zip › cells-2457482-supplementary.pdf]

Supplementary Table S1. Schedule of the implementation of stressors throughout the eight weeks of the CUMS protocol.

**Acclimatization week (No CUMS)**							
**Week 1**							
**Schedule**	**Monday**	**Tuesday**	**Wednesday**	**Thursday**	**Friday**	**Saturday**	**Sunday**
Morning (7am -12 am)		Empty box	Movement restriction	Cold (5˚C)	Overcrowding	Sucrose preference test	Continuous light
Evening (12am-7pm)	Predator odor	Rotation box	Wet box	Tilted box (45˚)	Rotating box/ Food deprivation		Continuous light/ Wet box
Night (7pm-7 am)	Overcrowding	White noise		Wet bed	Food deprivation		Continuous light
**Week 2**							
**Schedule**	**Monday**	**Tuesday**	**Wednesday**	**Thursday**	**Friday**	**Saturday**	**Sunday**
Morning (7am -12 am)	Movement restriction	Empty box	Continuous light			Sucrose preference test	White noise
Evening (12am-7pm)	Tilted box (45˚)	Cold (5˚C)	Continuous light/Rotation box	Wet box	Food deprivation		White noise
Night (7pm-7 am)	Wet bed		Continuous light	Strobe light	Food deprivation		Cold (5˚C)
**Week 3**							
**Schedule**	**Monday**	**Tuesday**	**Wednesday**	**Thursday**	**Friday**	**Saturday**	**Sunday**
Morning (7am -12 am)	Predator odor	Strobe light	Rotation box	Strobe light	Tilted box (45˚)	Sucrose preference test	Continuous light/ Overcrowding
Evening (12am-7pm)	Empty box	Wet box	Movement restriction	Overcrowdi ng	Food deprivation		Continuous light
Night (7pm-7 am)		White noise	Cold (5˚C)		Food deprivation		Continuous light
**Week 4**							
**Schedule**	**Monday**	**Tuesday**	**Wednesday**	**Thursday**	**Friday**	**Saturday**	**Sunday**
Morning (7am -12 am)	Continuous light	Strobe light	Empty box	Predator odor	Overcrowding	Sucrose preference test	Cold (5˚C)
Evening (12am-7pm)	Continuous light/ Tilted box (45˚)	Cold (5˚C)	Movement restriction		Food deprivation		Wet bed
Night (7pm-7 am)	Continuous light	White noise	Rotation box	White noise	Food deprivation		Wet bed
**Week 5**							
**Schedule**	**Monday**	**Tuesday**	**Wednesday**	**Thursday**	**Friday**	**Saturday**	**Sunday**
Morning (7am -12 am)	Cold (5˚C)	Empty box	Movement restriction	Tilted box (45˚)	Rotation box		Continuous light /Overcrowdin g

Evening (12am-7pm)	Predator odor	Rotation box	Overcrowding	Cold (5˚C)	Food deprivation	Sucrose preference test	Continuous light /Wet box
Night (7pm-7 am)		White noise		Wet bed	Food deprivation		Continuous light
**Week 6**							
**Schedule**	**Monday**	**Tuesday**	**Wednesday**	**Thursday**	**Friday**	**Saturday**	**Sunday**
Morning (7am -12 am)	Movement restriction	Empty box	Rotation box	Strobe light	Rotation box	Sucrose preference test	White noise
Evening (12am-7pm)	Tilted box (45˚)	Cold (5˚C)	Wet box	Predator odor	Cold (5˚C)/Food deprivation		White noise
Night (7pm-7 am)	Wet bed	White noise		Cold (5˚C)	Food deprivation		Cold (5˚C)
**Week 7**							
**Schedule**	**Monday**	**Tuesday**	**Wednesday**	**Thursday**	**Friday**	**Saturday**	**Sunday**
Morning (7am -12 am)	Predator odor	Strobe light	Rotation box	Tilted box (45˚)	Empty box	Sucrose preference test	Continuous light /Overcrowding
Evening (12am-7pm)		Wet box	Movement restriction		Food deprivation		Continuous light
Night (7pm-7 am)	Empty box	White box	Cold (5˚C)	Wet bed	Food deprivation		Continuous light
**Week 8**							
**Schedule**	**Monday**	**Tuesday**	**Wednesday**	**Thursday**	**Friday**	**Saturday**	**Sunday**
Morning (7am -12 am)	Cold (5˚C)	Strobe light	Empty box	Predator odor	Overcrowding	Sucrose preference test	Continuous light
Evening (12am-7pm)	Tilted box (45˚)	Rotation box	Movement restriction		Food deprivation		Continuous light
Night (7pm-7 am)	Overcrowding	Movement restriction	Cold (5˚C)	White noise	Food deprivation		Continuous light/Wet bed
